# Preliminary Analysis of Bilberry NaDES Extracts as Versatile Active Ingredients of Natural Dermocosmetic Products: In Vitro Evaluation of Anti-Tyrosinase, Anti-Hyaluronidase, Anti-Collagenase, and UV Protective Properties

**DOI:** 10.3390/plants14152374

**Published:** 2025-08-01

**Authors:** Milica Martinović, Ivana Nešić, Ana Žugić, Vanja M. Tadić

**Affiliations:** 1 Department of Pharmacy, Faculty of Medicine, University of Niš, Boulevard Dr. Zorana Djindjića 81, 18108 Nis, Serbia; milica.martinovic@medfak.ni.ac.rs (M.M.); ivana.nesic@medfak.ni.ac.rs (I.N.); 2 Department for Pharmaceutical Research and Development, Institute for Medicinal Plant Research “Dr. Josif Pančić”, Tadeuša Koscuška 1, 11000 Belgrade, Serbia; azugic@mocbilja.rs

**Keywords:** sun protection, anti-tyrosinase, anti-hyaluronidase, anti-collagenase, NaDES, anti-age, enzyme, inhibition, bilberry

## Abstract

Bilberry (*Vaccinium myrtillus* L.) fruits represent the recognized wellspring of bioactive compounds with various documented bioactivities. Although bilberry leaves are often treated as industrial by-products, they also represent a valuable source of phytochemicals with potential dermocosmetic applications. In this study, extracts of bilberry fruits and leaves were prepared using both conventional solvents (water and 50% ethanol) and natural deep eutectic solvents (NaDES) as green, biodegradable alternatives. The aim of this study was to examine the UV protective activity and inhibitory potential of those extracts against some enzymes (tyrosinase, hyaluronidase, collagenase) that are important in terms of skin conditioning and skin aging. The results of in vitro tests have shown the superiority of NaDES extracts compared to conventional extracts regarding all tested bioactivities. In addition, bilberry leaves extracts were more potent compared to fruit extracts in all cases. The most potent extract was bilberry leaf extract made with malic acid–glycerol, which exhibited strong anti-tyrosinase (IC_50_ = 3.52 ± 0.26 mg/mL), anti-hyaluronidase (IC_50_ = 3.23 ± 0.30 mg/mL), and anti-collagenase (IC_50_ = 1.84 ± 0.50 mg/mL) activities. The correlation analysis revealed correlation between UV protective and anti-tyrosinase, UV protective and anti-collagenase as well as between anti-hyaluronidase and anti-collagenase activity. UV protection and anti-tyrosinase activity correlated significantly with chlorogenic acid and hyperoside contents in extracts. The extracts with the best activities also demonstrated a good safety profile in a 24 h in vivo study on human volunteers.

## 1. Introduction

Healthy skin is elastic and radiant, and has youthful and fresh appearance, while damaged skin looks pale, tired, and dehydrated, which is especially evident in the thinnest areas, such as areas around the eyes. Over recent decades, the cosmetic industry has undergone significant changes, driven by an improved understanding of healthy skin physiology and advancements in research methodologies. As a result, there has been progress in the discovery of new active ingredients and carriers, based on well-studied mechanisms of action, leading to noticeable advancements in the development of modern cosmetic and dermocosmetic products. In addition, there is a growing emphasis on the sustainability, biodegradability, and environmental compatibility of cosmetic products as a new trend in the industry, aligning with rising consumer awareness about health and ecological impact. The increasing demand for “green” and “natural” cosmetics stems not only from environmental concerns but also from safety issues, as conventional cosmetic ingredients such as UV filters, fragrances, and preservatives are associated with adverse effects, including allergic contact dermatitis [1].

As a rich source of bioactive substances, such as flavonoids, tannins, anthocyanins, carotenoids, and vitamins, plant extracts exhibit significant antioxidant properties. Furthermore, they serve as functional ingredients that can have additional dermocosmetic effects, including anti-inflammatory, antibacterial, UV protective, hypopigmenting, emollient, and moisturizing effects. Their multifunctionality makes them valuable ingredients in formulations designed to protect the skin from environmental stressors, combat aging, hydrate dry skin, treat acne, and improve overall skin condition [2].

Bilberry (*Vaccinium myrtillus* L., Ericaceae) is a low-growing shrub abundant in phenolic compounds with known anti-inflammatory, antioxidant, and metabolic health effects. While bilberry fruits (BFs) are commonly used in traditional medicine, the leaves (BLs) and seeds are typically discarded as industrial by-products. However, recent studies have revealed that BLs contain high level of bioactive phytochemicals and exhibit significant antioxidant activity. Moreover, they show promising skin-rejuvenating activity by enhancing the expression of genes in fibroblasts and stimulating synthesis of hyaluronic acid and gluthatione [3]. As for dermocosmetic purposes, the use of BF is traditionally associated with antiseptic, adstrigent, and tonic activities [4]. The studies have confirmed skin-lightening, anti-inflammatory, anti-aging, antioxidant, UV protective, antimicrobial and chemo-preventive effects on BF [5]. In addition, both Vaccinium Myrtillus Fruit Extract and Vaccinium Myrtillus Leaf Extract are listed in the Cosing Database as skin-conditioning agents [6].

In our previous research, we prepared extracts of BF and BL using natural deep eutectic solvents (NaDES), green solvents formed by combination of sugars, amino acids, and other plant metabolites, resulting in a mixture with a melting point lower than each of their individual components. Such extracts are environmentally friendly, biodegradable, safer, richer and more effective compared to extracts obtained using conventional solvents. Previous reports have shown that NaDES can enhance solubility, stability, bioavailability, and even dermal delivery of active compounds from the extracts [7]. In addition, NaDES have been used in topical formulations, like gels, creams of emulgels, and improved drug loading and skin penetration [8].

In this study, NaDES composed of alpha-hydroxy acids (AHAs) and polyols were used: malic acid + glycerol (MG), tartaric acid + sorbitol (TS), and citric acid + sorbitol (CS). NaDES not only facilitate the extraction of active compounds but also contribute beneficially to the biological activity of the final product—unlike organic solvents, which are toxic, or ethanol, which can irritate the skin, or water, which often requires the addition of preservatives. Our previous study indicated that NaDES extracts had higher polyphenol content and exhibited better antioxidant activity compared to water and ethanol extracts [9].

Numerous enzymes such as tyrosinase, hyaluronidase, collagenase, elastase, and others are present in the skin, and contribute to its structure and function. When overexpressed, they can contribute to skin aging and pigmentation disorders. Their activity may increase naturally over time or be induced by external stress factors such as UV radiation, making their inhibition a promising anti-aging strategy [10]. In addition, excessive exposure to UV radiation may lead to accelerated skin aging due to the remodeling of the skin immune system and damaging of DNA and skin protein structures. UV rays may also cause local inflammation and activation of pro-inflammatory cytokines [11]. Therefore, photoprotection is an important aspect of proper skin care [12].

The aim of this study was to evaluate the in vitro inhibitory effects of NaDES-based extracts from bilberry leaves and fruits against tyrosinase, hyaluronidase, and collagenase—key enzymes involved in melanin production and degradation of extracellular matrix components like hyaluronic acid and collagen. Additionally, the goal of the study was to examine the UV protective effect of NaDES extracts and to compare all the determined activities between NaDES extracts and conventional extracts (water and ethanol), as well as between BFs and BLs, taking into account that the BL is mostly obtained as BF processing waste. All assessed biological activities were evaluated with respect to chemical composition of the prepared extracts. Finally, the aim was also to determine whether there is a correlation between obtained results of the biological activities and chemical composition of the extracts.

## 2. Results and Discussion

**Figure 1:**
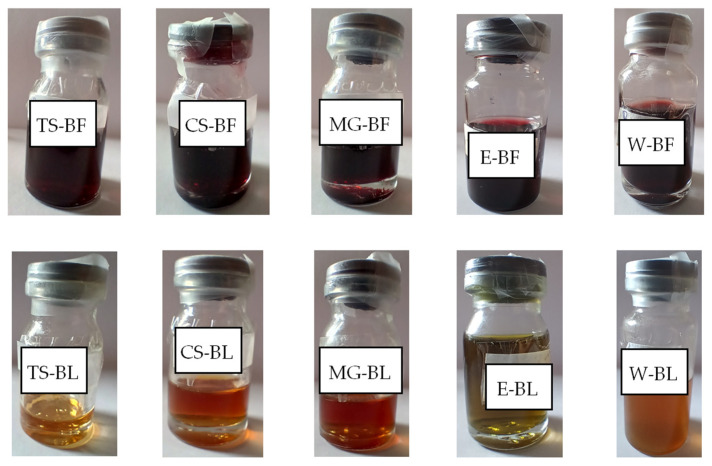
The bilberry fruits (BF) and leaves (BL) extracts prepared using NaDES as green extraction solvents (TS, CS, MG) and water (W) and 50% ethanol (E) as conventional solvents, used in this study.

In the experiments presented in this paper, ten extracts were prepared (Figure 1) using ultrasound-assisted extraction (UAE) as the contemporary extraction method, which is commonly employed with NaDES as extraction solvents. This extraction method is based on high-frequency ultrasound that causes cavitation and enhances cell wall permeability, thus increasing mass transfer. In this way, less energy is used, the extraction time is shortened, and carbon emission is lowered. In addition, obtained extracts have a higher yield of bioactive compounds [13].

Two groups of the prepared extracts—BF (bilberry fruit extracts) and BL (bilberry leaves extracts) were obtained using five extraction solvents: water (W) and 50% ethanol (E) as conventional solvents and malic acid–glycerol (1:2 mol/mol) (MG), tartaric acid–sorbitol (1:2 mol/mol) (TS), and citric acid–sorbitol (1:2 mol/mol) (CS) as NaDES.

Even though solvents that were used were transparent liquids (E, W, TS, CS, MG), the obtained extracts were colored (Figure 1). Unlike NaDES extracts that were transparent, ethanol and especially water extracts were not.

There are several previous reports of NaDES used as extraction solvents for BF extraction. For instance, ten different choline chloride-based NaDES were prepared, and choline chloride–sorbitol (1:1 mol/mol) was selected as the best choice for anthocyanin extraction and obtaining extracts with promising antioxidant and antimicrobial activity [14]. In addition, other NaDES made of choline chloride–glycerol–citric acid (1:4:1 mol/mol/mol) were as efficient as conventional organic solvent (methanol–water–formic acid) for anthocyanin extraction [15]. Choline chloride-based NaDES were also used for the extraction of bilberry peels [16]. However, choline chloride is restricted for use in cosmetics due to Regulation (EC) No 1223/2009, so other NaDES should be used for preparing extracts intended for cosmetic purposes [17].

When it comes to BL, in previous studies, 18 NaDES were prepared and tested for BL extraction. The best extraction was achieved using following NaDES: lactic acid–sodium acetate–water (3:1:2), and choline chloride–oxalic acid (1:1) [18].

To the best of our knowledge, so far NaDES extracts of BF and BL were not tested for UV protective, anti-tyrosinase, anti-hyaluronidase, and anti-collagenase activity.

### 2.1. HPLC Analysis

The predominant phenolic compounds identified by HPLC analysis in tested extracts included flavonols (rutin, hyperoside, quercetin-3-*O*-glucoside), hydroxycinnamic acids (chlorogenic acid), flavanols (epicatechin, procyanidin B2), *p*-hydroxybenzoic acids (protocatechuic acid), and anthocyanins (delphinidin-3-*O*-glucoside, cyanidin-3-*O*-glucoside, cyanidin-3-*O*-galactoside). The results of the chemical (HPLC) analysis of bilberry fruit and leaves extracts were presented in Table 1.

### 2.2. UV Protective Activity

Ultraviolet (UV) radiation is one of the most impactful external factors—collectively referred to as exposomes—that contribute to skin damage and accelerate the aging process. Even the term “photoaging” was established to emphasize this relationship. Since photoaging affects both epidermis and dermis, regular use of sunscreens is recommended for delaying this process [19].

There are various methods for in vitro assessment of UV protective activities of herbal extracts that are commonly used due to simplicity, cost-effectiveness, as well as ethic and safety concerns. In this study, we applied a dilution method to measure absorbance of diluted solutions and calculate the Sun Protection Factor (SPF) using Mansur Equation (1) [20].

**Figure 2:**
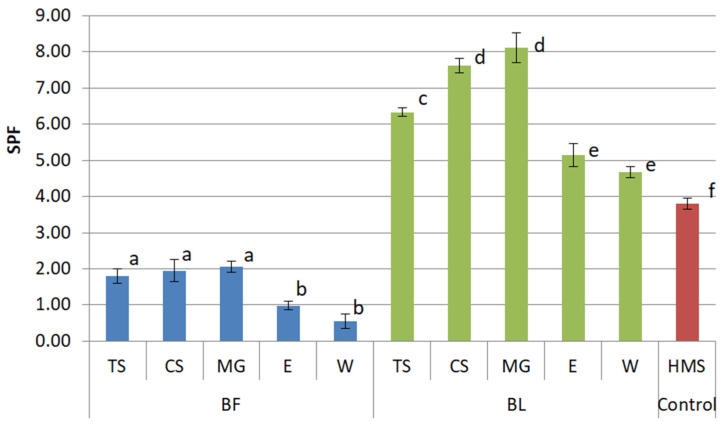
UV protective activity (SPF) of tested bilberry fruits (BF) and leaves (BL) extracts prepared using NaDES as extraction solvents (TS, CS, MG) and water (W) and 50% ethanol (E) as conventional solvents. As control, 8% homosalate (HMS) was used. (Different letters indicate statistically significant differences (*p* < 0.05). Any extracts that share the same letter are not significantly different from each other).

The results of our study revealed that NaDES extracts exhibited higher UV protective potential compared to water and ethanol extracts (*p* < 0.05) (Figure 2). Sole solvents (W, E, TS, CS, MG) showed no significant absorption in UV spectrum, so they were not portrayed in Figure 2.

The enhanced efficacy of BF extracts prepared with NaDES can be attributed to the presence of anthocyanins, which are responsible for UV protective potential of BF [5]. Anthocyanins detected in tested extracts were delphinidin-3-*O*-glucoside, cyanidin-3-*O*-galactoside, and cyanidin-3-*O*-glucoside (Table 1). Unlike water and ethanol extracts, where they were present in traces, they were detected in NaDES extracts (MG, TS, and CS) in notable amounts (Table 1), which might explain why NaDES BF extracts (MG-BF, TS-BF, and CS-BF) had similar SPFs, higher than the SPF of water and ethanol BF extracts (*p* < 0.05).

Previous studies have shown that BF extracts can protect human keratinocytes (HaCaT cells) from UVA-induced lipid peroxidation and glutathione depletion [21]. Furthermore, BF reduced reactive oxygen species (ROS) generation and apoptosis induced by UVA rays as well as UVB rays mediated lipid peroxidation in HaCaT keratinocytes [22,23]. SPF values for bilberry extracts reported in the literature were different depending on the solvent used. While lipophilic chloroform extract showed SPF 3.6 [24], which is close to the SPF measured in our study, some other authors even measured a 10 times higher SPF (34.5) [25]. However, a high value of SPF like this is unusual for herbal extracts.

Interestingly, the results of our study also indicated that leaves have more potent UV protection potential compared to fruits, as all BL extracts exhibited higher SPF values and a higher phenolic content than their corresponding BF extracts (*p* < 0.05). The highest measured SPF in the study was detected for MG-BL (8.12 ± 0.42) followed by CS-BL (7.62 ± 0.20).

To the best of our knowledge, there are no studies where the UV protective activity of BL was estimated. However, there are studies that indicate the protective role of flavonoids and cinnamic acids against UV radiation in plants [26] which might explain why BL extracts richer in phenolic compounds (Table 1) have higher SPF values compared to BF.

To sum up, the SPF calculated using the in vitro dilution method and Mansur Equation (1) showed higher UV protective potential of bilberry leaves compared to bilberry fruits. In addition, NaDES extracts showed significantly higher SPF values than water and ethanol extracts (*p* < 0.05), likely due to a higher content of anthocyanins and other phenolic compounds. The extract with the highest calculated SPF was MG-BL.

### 2.3. Anti-Tyrosinase Activity

Tyrosinase is an enzyme that plays important role in melanogenesis, which, to an excessive extent, leads to abnormal melanin accumulation, and thus the formation of various types of freckles, age spots, hyperpigmentation, and melasmas. Although melanin serves as a natural photoprotective pigment and natural skin UV filter, its overproduction leads to undesirable aesthetic effects [10].

The anti-tyrosinase assay used in the study was based on the formation of characteristically colored dopachrome, resulting from the reaction between L-DOPA and mushroom tyrosinase [10]. The positive control used in the study was ascorbic acid (IC_50_ = 0.34 ± 0.18 mg/mL).

All BL extracts have shown better tyrosinase inhibitory activity compared to corresponding BF extracts (*p* < 0.05). The reason for this might be higher phenolic content in BL extracts. For instance, among phenolic compounds identified by HPLC analysis, chlorogenic acid as tyrosinase inhibitor [27] was identified in an almost 20 times greater amount in BL extracts than in BF extracts (Table 1). Also, hyperoside, identified as an tyrosinase inhibitor by Li et al. [28], was detected in higher concentrations in BL extracts (from 1.32 ± 0.11 to 3.31 ± 0.10 mg/g) than in BF extracts (from 0.15 ± 0.03 to 0.57 ± 0.06 mg/g) (Table 1). In addition, other compounds known for tyrosinase inhibitory activity were identified in significant amounts in BL extracts, like quercetin-3-*O*-glucoside [29], rutin [30], and procyanidin B2 [31].

**Figure 3:**
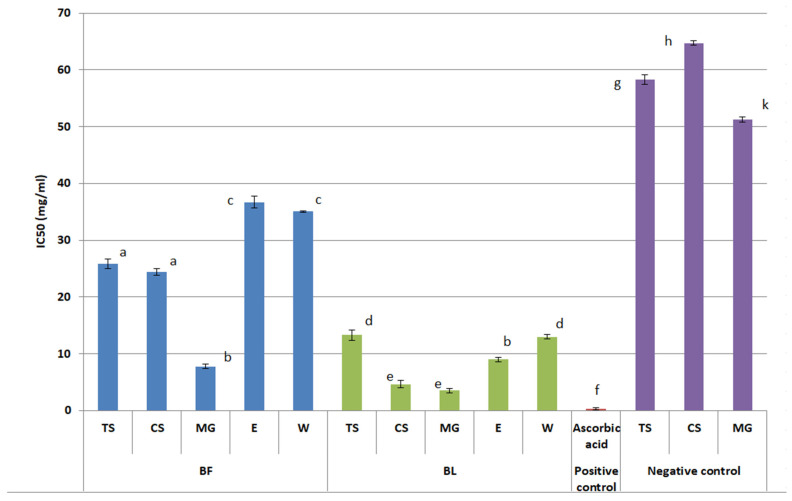
Anti-tyrosinase activity (IC_50_ values) of tested bilberry fruits (BF) and leaves (BL) extracts prepared using NaDES as extraction solvents (TS, CS, MG) and water (W) and 50% ethanol (E) as conventional solvents. As positive control, ascorbic acid was used, while clean solvents (TS, CS, MG, W, and E were used as negative control. W and E showed no anti-tyrosinase activity). (Different letters indicate statistically significant differences (*p* < 0.05). Extracts that share the same letter are not significantly different from each other).

NaDES extracts exhibited superior tyrosinase inhibitory activity compared to conventional extracts, with the exception of TS-BL (Figure 3). Among all the extracts, the best inhibitory activity was observed for MG-BL whose IC_50_ was 3.52 ± 0.26 mg/mL. Within both the BF and BL groups, MG-extracted samples consistently showed the greatest inhibitory effect (*p* < 0.05).

While W and E as negative controls did not inhibit tyrosinase, MG, TS, and CS showed low inhibitory activity with IC_50_ values higher compared to the IC_50_ values of corresponding extracts (Figure 3). So far, previous studies have shown that malic and citric acid have tyrosinase-inhibitory potential [32,33]. Additionally, glycerol has been shown to enhance tyrosinase inhibition when used with flavonoid complexes, as demonstrated in *Sophora japonica* L. extracts [34].

The hypopigmentation effects of bilberry extracts have been previously reported. For instance, some commercially manufactured juices from BF have shown comparable tyrosinase inhibitory potential at the concentration of 1 mg/mL as 0.02 mg/mL kojic acid [35]. Another study showed that water, methanol, methanol–water (1:1, *v/v*), and acetone–water (1:1, *v/v*) bilberry fruit extracts inhibited less than 50% of tyrosinase activity at 10 mg/mL [36], suggesting that the NaDES approach used in the present study yielded more potent inhibitory profiles.

Overall, the tyrosinase inhibitory assay revealed that all BL extracts showed significantly stronger tyrosinase inhibition than BF extracts and that the NaDES extracts (especially MG-BL) exhibited the most potent inhibitory activity.

### 2.4. Anti-Hyaluronidase Activity

Hyaluronic acid is the main component of the extracellular matrix and probably the world’s most famous glycosaminoglycan. Owing to its remarkable hygroscopic nature—it can retain up to 1000 times its weight in water—hyaluronic acid plays a crucial role in maintaining skin hydration in both the epidermis and dermis and preserving the youthful and healthy being of the skin. It consists of D-glucuronic acid and D-N-acetylglucosamine linked via β-1,3 and β-1,4 bonds. The enzyme hyaluronidase, which is naturally present in the human body, is responsible for breaking down this macromolecule into monosaccharides. Excessive hyaluronidase activity is associated with reduced skin moisture, loss of elasticity, and the formation of wrinkles. Therefore, the inhibition of hyaluronidase represents significant tool in anti-aging strategies [37,38].

The hyaluronidase inhibition assay is based on a turbidimetric method, which quantifies the turbidity caused by residual hyaluronic acid that remains undegraded by hyaluronidase in the presence of an enzyme inhibitor (herbal extract). Therefore, greater turbidity corresponds to stronger hyaluronidase inhibition [10].

**Figure 4:**
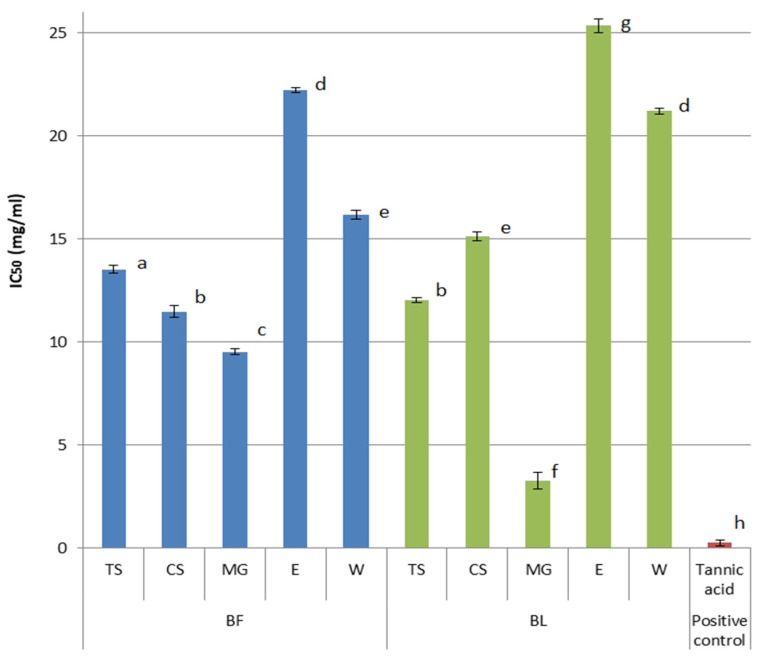
Anti-hyaluronidase activity (IC_50_ values) of tested bilberry fruits (BF) and leaves (BL) extracts prepared using NaDES as extraction solvents (TS, CS, MG) and water (W) and 50% ethanol (E) as conventional solvents. As control, tannic acid was used. (Different letters indicate statistically significant differences (*p* < 0.05). Extracts that share the same letter are not significantly different from each other).

The hyaluronidase inhibitory activity was portrayed in Figure 4. The best hyaluronidase inhibitory activity was observed for BL-MG (IC_50_ = 3.23 ± 0.30 mg/mL). Across both BF and BL groups, all NaDES extracts demonstrated lower IC_50_ values (*p* < 0.05) than their water or ethanol counterparts (*p* < 0.05), indicating enhanced enzyme inhibition. The components identified in the extracts (Table 1) that might have contributed to this activity, as suggested in previous investigations, were chlorogenic acid [39], rutin [40,41], quercetin-3-*O*-glucoside [41], epicatechin [42], and protocatechuic acid [43],

Among tested solvents, besides water and 50% ethanol, NaDES exhibited very low inhibiting activity against hyaluronidase (359.83–439.05 mg/mL) indicating minimal influence on the overall activity of the extracts. To the best of our knowledge, there are no available data on the influence of AHAs or glycerol on hyaluronidase. However, some studies report that sorbitol can stabilize hyaluronic acid and enhance its resistance to enzymatic degradation [44]

Among all extracts, those prepared with 50% ethanol exhibited the weakest activity, with IC_50_ values were significantly higher than those of NaDES extracts. Notably, the BL ethanol extract showed a nearly eightfold higher IC_50_ value compared to the MG extract.

Our extracts showed better hyaluronidase inhibitory properties than the BF water–acetone extract from the literature data, which was able to inhibit 90% of enzyme’s activity at concentration 50 mg/mL [36]. However, our extracts had higher IC_50_ values compared to the positive control, tannic acid (IC_50_ = 0.026± 0.02 mg/mL).

Generally speaking, the turbidimetric assay revealed hyaluronidase inhibitory activity of bilberry fruit (BF) and leaf (BL) extracts made with all extraction solvents used in the study. While ethanol extracts had the weakest activity, NaDES extracts, especially BL-MG, had lower IC_50_ values and were more active in terms of hyaluronidase inhibition. Compared to the literature data, these extracts demonstrated stronger inhibitory activity than some previously tested BF extracts but were still less effective than the positive control, tannic acid.

### 2.5. Anti-Collagenase Activity

The collagenase inhibition assay in this study was based on the spectrophotometric method developed by Zhang et al. [45], which is simple, reliable, and economic. Unlike other methods that use radiolabeled or fluorescence-labeled collagen, it does not require specialized instruments or generate radioactive waste. This assay is based on the colorimetric ninhydrin reaction with free amino acids released after the degradation of collagen, since the absorbance of obtained “Ruhemann’s purple” at 570 nm is proportional to the concentration of free amino acids [45].

Collagen, a key protein in the extracellular matrix, makes up nearly 75% of the skin’s dry weight. As the most abundant skin structural protein, it plays a crucial role in preserving not only the skin’s structure and function but also its youthful appearance. Collagen provides strength and stability to skin tissue by enabling collagen fibrils to slide and realign, allowing the skin to stretch and move without losing its integrity or sustaining damage. Collagenase is the enzyme present in skin responsible for the degradation of collagen, a process that naturally occurs as a consequence of aging or is stimulated by the influence of external factors like UV radiation or smoking. As a result, collagen is being degraded and the skin has a wrinkled and toneless look [46]. Therefore, screening for new natural collagenase inhibitors is an important strategy in anti-aging skincare research [47].

The results of the collagenase inhibition assay were presented in Table 2. The extracts of BL had lower IC_50_ values than BF extracts, indicating that leaves demonstrated better collagenase inhibition activity compared to the fruits (*p* < 0.05). The best anti-collagenase activity was observed for MG-BL, the extract that also exhibited the best UV protective, anti-hyaluronidase, and anti-tyrosinase activity.

Among phenolic components identified in BF and BL extracts (Table 1), those with known collagenase inhibition potential were rutin [40], hyperoside [48], protocatechuic acid [43], chlorogenic acid [49], and quercetin [50].

Furthermore, NaDES extracts showed better inhibitory potential than water and ethanol extracts, whose IC_50_ values were very high, indicating that NaDES components themselves contribute to collagenase inhibition. Malic acid, tartaric acid, and citric acid are commonly used AHAs—weak organic acids, with hydroxyl group attached to the carbon in alpha position [51]. The study on the human skin of Japanese subjects revealed that citric acid can induce the proliferation of collagen I and procollagen II. The same was observed for other AHAs—glycolic and lactic acid [52]. Additionally, glycerol as a component of used NaDES was studied as extraction solvent for obtaining extracts with notable collagenase inhibitory activity [47]. The presence of glycerol in extracts can prevent the denaturation of collagen [53].

The results of our study indicated that none of the solvents used in the study expressed collagenase inhibition activity (Table 2). However, due to the chemical interactions between NaDES solvents and compounds in extracts, it is possible that they might have contributed to the observed extracts’ activity. Several studies support the role of NaDES in enhancing collagenase inhibition. For example, choline chloride–glycerol and choline chloride–urea extracts of green tea showed excellent collagenase inhibition [54]. Proline–glycerol *Jasione montana* extract, rich in phenolic compounds, was an even stronger collagenase inhibitor compared to gallic acid [55]. Grape pomace extract obtained via the betaine–glucose mixture demonstrated better collagenase inhibition compared to ethanol extract [56].

Altogether, as observed in the other tests, the bilberry leaves were more potent than the fruits. However, unlike other performed assays, in case of collagenase inhibition, water and ethanol extracts exhibited low activity. The most potent inhibitor was MG-BL, which also was marked as the best candidate in terms of UV protection, tyrosinase, and hyaluronidase inhibition.

### 2.6. Correlation Study

The correlation analysis was performed in order to explore relationships between chemical composition (content of chlorogenic acid and hyperoside, as the compounds detected by HPLC in all the tested extracts) and biological activities investigated in the paper—UV protective (SPF), anti-tyrosinase (Tyr), anti-hyaluronidase (Hyal), and anti-collagenase (Col) activities.

The results of correlation analysis were presented using a correlation matrix (Table 3), where the color gradient ranged from red as a sign of the strongest positive correlation (r = 1) to white (no correlation, r = 0) and blue as signs of the strongest negative correlation (r = −1). Negative correlation was important since some data (anti-tyrosinase, anti-hyaluronidase, anti-collagenase activity) are presented as IC_50_ values, where a lower value indicates higher activity.

According to the correlation matrix, there was statistically significant strong correlation between the SPF and chlorogenic acid content, as well as between the SPF and hyperoside content, indicating that the SPF value might be proportional to the content of phenolic compounds. Additionally, the SPF was inversely correlated with IC_50_ values for anti-tyrosinase and anti-collagenase activities, suggesting that extracts with higher UV protection potential also possess greater enzyme-inhibitory effects. Anti-tyrosinase activity also significantly correlated with content of both chlorogenic acid and hyperoside. Furthermore, correlation between hyaluronidase and collagenase activity was observed.

### 2.7. In Vivo Safety Assessment

For the purpose of assessing the irritant potential of the extracts, the method of extract application under occlusion (so-called patch testing) is commonly used, as occlusion is expected to enhance the effects on the skin. Patches soaked with the extracts are usually applied for a period of 24 to 48 h, and the measured values of biophysical parameters are compared with the baseline values [57].

The in vivo safety study on human volunteers, which lasted for 24 h, revealed that the tested extracts (MG-BL and CS-BF) did not cause any adverse skin reactions. As part of this study, parameters were measured at four sites on the forearms of volunteers, with one site marked as an non-treated control site (NC) and one as an non-treated control site under occlusion (NCO), while filter papers soaked with the chosen extracts (MG-BL and CS-BF) were applied to the remaining two sites, which were then covered with occlusion that consisted of Parafilm^®^ and Hartmann Omnifix^®^ E elastic adhesive tape.

The results showed statistically significant changes in almost all parameters at the NCO site, while parameters did not significantly change at NC, indicating that occlusion caused changes in skin parameters. At NCO, a statistically significant decrease in pH was observed, as well as an increase in TEWL (transepidermal water loss), EC (electrical capacitance), and EI (erythema index).

**Figure 5:**
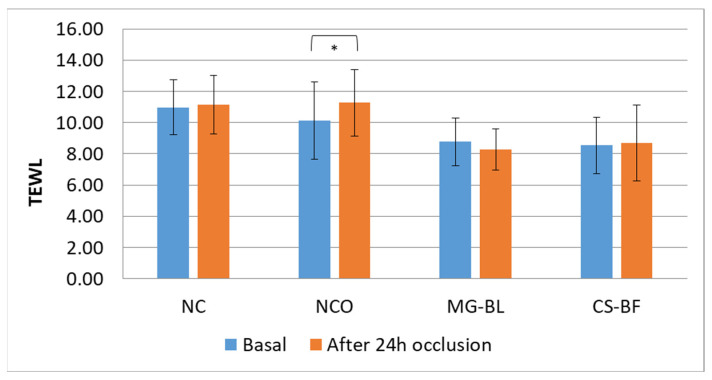
Average basal TEWL (transepidermal water loss) values as well as values measured after 24 h occlusion in an in vivo safety study for determining the irritant potential of selected plant extracts (MG-BL and CS-BF), non-treated control (NC), and non-treated control under occlusion (NCO). Values are presented as mean ± standard deviation, while statistically significant differences are indicated by the symbol * (*p* < 0.05).

**Figure 6:**
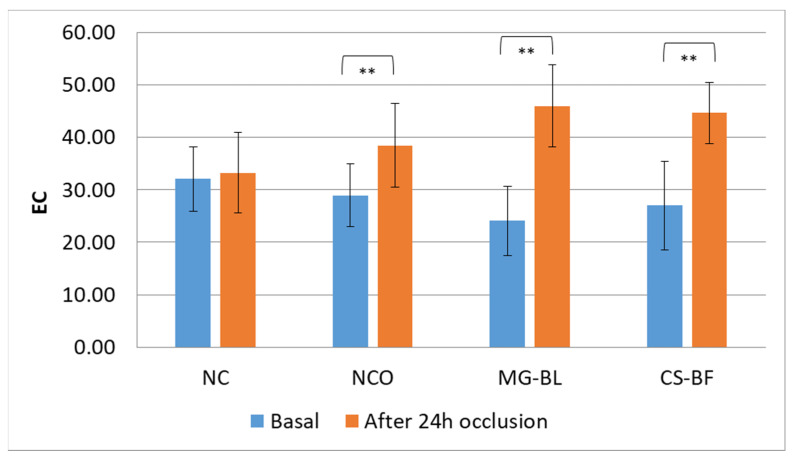
Average basal EC (electrical capacitance) values as well as values measured after 24 h occlusion in an in vivo safety study for determining the irritant potential of selected plant extracts (MG-BL and CS-BF), non-treated control (NC), and non-treated control under occlusion (NCO). Values are presented as mean ± standard deviation, while statistically significant differences are indicated by the symbol ** (*p* < 0.01).

An increase in TEWL at the NCO (Figure 5) may indicate a slight disruption of the skin barrier or might be a consequence of elevated hydration under occlusion, since occlusion on the skin surface prevents water evaporation, thereby causing its accumulation in the intercellular spaces of the stratum corneum and swelling of the corneocytes. Consequently, an increase in both TEWL and skin hydration often occurs in occluded sites [58]. However, considering that a statistically significant increase in hydration was observed at all occluded sites (NCO, MG-BL, and CS-BF) (Figure 6), it cannot be concluded that the increase in TEWL at the NCO site is solely the result of increased hydration. In addition, NaDES extracts did not cause elevation of TEWL, indicating that they did not have a negative influence on the skin barrier.

**Figure 7:**
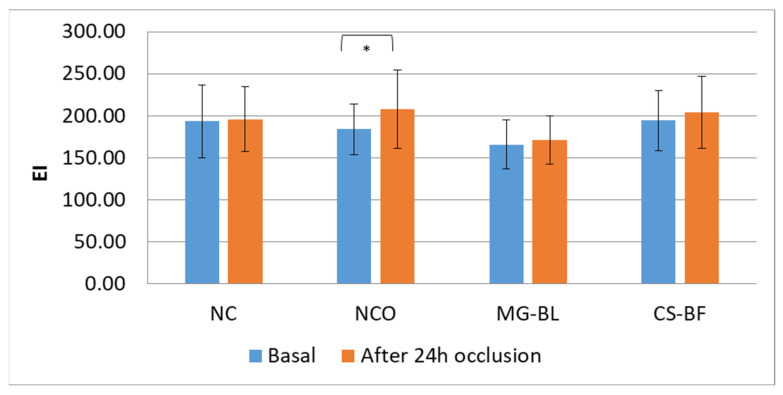
Average basal EI (erythema index) values as well as values measured after 24 h occlusion in an in vivo safety study for determining the irritant potential of selected plant extracts (MG-BL and CS-BF), non-treated control (NC), and non-treated control under occlusion (NCO). Values are presented as mean ± standard deviation, while statistically significant differences are indicated by the symbol * (*p* < 0.05).

The investigation of EI (Figure 7) pointed out that there was a statistically significant increase in EI values only at the NCO site, which supported the assumption of a mild disruption of the skin barrier and mild irritation due to occlusion.

On the other hand, at the sites under occlusion treated with both NaDES extracts (MG-BL and CS-BF), no increase in TEWL or statistically significant increase in EI was observed, leading to the conclusion that the tested extracts, in some way, reduced the irritation observed at the NCO site. Thus, not only did they show no irritant potential after one day of occlusion, but the results of the in vivo study might indicate prospective anti-irritant potential.

**Figure 8:**
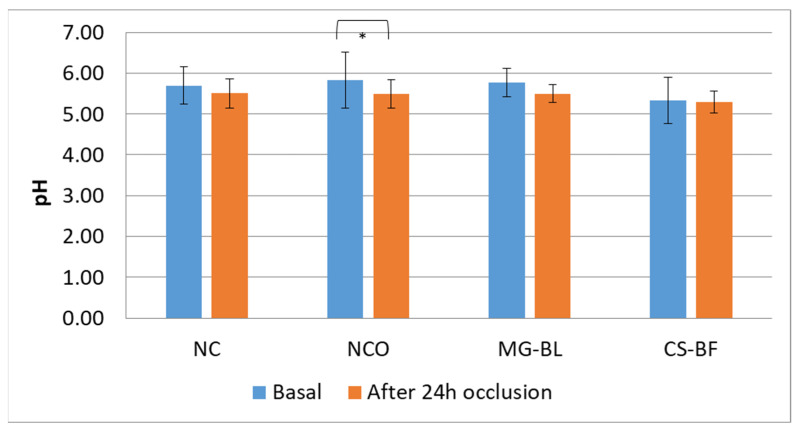
Average basal pH values as well as values measured after 24 h occlusion in an in vivo safety study for determining the irritant potential of selected plant extracts (MG-BL and CS-BF), non-treated control (NC), and non-treated control under occlusion (NCO). Values are presented as mean ± standard deviation, while statistically significant differences are indicated by the symbol * (*p* < 0.05).

In addition, no statistically significant changes in pH values at the other tested sites (Figure 8) were observed, which is important since pH may signalize changes in skin barrier function [59].

Overall, the non-invasive in vivo study on human volunteers highlighted that tested NaDES extracts (MG-BL and CS-BF) under occlusion did not disrupt basal values of skin parameters (pH, TEWL, EC, and EI).

## 3. Materials and Methods

### 3.1. Materials

Herbal materials used in this study were bilberry fruits and bilberry leaves (*Myrtilli fructus*, *Myrtilli folium*, *Vaccinium myrtillus* L., Ericaceae), whose identity was confirmed at Herbarium of Faculty of Pharmacy, University of Belgrade (Belgrade, Serbia) where the vouchers specimens were deposited (VML_0921).

Purified water, obtained from the Faculty of Medicine (University of Niš, Niš, Serbia) and 50% ethanol (*v/v*), prepared from 96% ethanol (Sigma-Aldrich, St. Louis, MO, USA), were used as conventional extraction solvents. For NaDES preparation tartaric acid and malic acid purchased from Centrohem (Stara Pazova, Serbia), as well as sorbitol and glycerol purchased from Comcen (Beograd, Serbia) were used.

Reference HPLC standards, chlorogenic acid, protocatechuic acid, delphinidin-3-*O*-glucoside, hyperoside, cyanidin-3-*O*-galactoside, cyanidin-3-*O*-glucoside, procyanidin B2, quercetin-3-*O*-glucoside, rutin, quercitrin, and epicatechin (purity ≥ 99%) were purchased from Extrasynthese (Genay, France).

Biological activities of extracts (UV protective, anti-tyrosinase, anti-collagenase, and anti-hyaluronidase activity) were assessed using following analytical grade reagents purchased from Sigma-Aldrich: ethanol (70%, *v/v*), collagenase, gelatin, hyaluronidase, hyaluronic acid, bovine serum albumin, and L-DOPA mushroom tyrosinase.

### 3.2. NaDES Extracts Preparation

NaDES tartaric acid–sorbitol (1:2 mol/mol) (TS), citric acid–sorbitol (1:2 mol/mol) (CS), and malic acid–glycerol (1:2 mol/mol) (MG), were prepared according to the procedure that involved heating the two individual components to 80 °C while continuously stirring them using a magnetic stirrer (IKAMAG, IKA, Verke, Staufen, Germany) for 30–60 min. Upon melting the mixture, a clear liquid is formed to which distilled water was added at 30% (*v/v*). The extraction conditions were adjusted after series of trial extractions.

Ultrasound-assisted extraction was conducted for 30 min at 50 °C in a sonication water bath (Gesellschaft fur Labortechnik, Burgwedel, Germany). The dried powdered plant material and solvents (TS, CS, MG, water or 50% ethanol) were mixed in mass ratio 1:20, commonly used proportion in the preparation of NaDES extracts [60,61,62]. All extracts were centrifuged after extraction and the supernatant was used for further work.

### 3.3. SPF Calculation Using Mansur Equation

The determination of UV absorption capacity was carried out using the dilution method based on application of Mansur Equation (1) [63].

(1) $$SPF=CF\times \sum_{290}^{320}EE\left(\lambda \right)\times I\left(\lambda \right)\times Abs(\lambda )$$

EE(λ) indicates the erythemal effect of solar radiation at a specific wavelength, I(λ) refer to the intensity of sunlight at a certain wavelength, while Abs(λ) is the absorbance measured by the spectrophotometer at a given wavelength. CF represents the correction factor. The values of EE(λ) × I(λ) are constants and were calculated by Sayre et al. [64].

The procedure involves making series of dilutions of extracts with ethanol until the final dilution of 0.2 mg/mL is achieved, after which the absorbance was measured at every 5 nm in the range of 290 to 320 nm using spectrophotometer Evolution 60, Thermo-Fisher Scientific (Waltham, MA, USA).

As positive control, 8% homosalate ethanol solution was used, whose SPF was 3.8 ± 0.18, while pure solvents (W, E, TS, CS, MG), were used as negative controls.

### 3.4. HPLC Analysis

For the qualitative and quantitative analysis of polyphenols, an Agilent 1200 HPLC system equipped with a photodiode array (PDA) detector was employed. The chromatographic conditions were set as in Table 4.

Sample preparation for chromatographic analysis was based on the dilution of extracts with deionized water to a concentration of 25 mg/mL and filtration using PTFE membrane filters prior to injection.

Compound identification was based on overlaying with retention times and UV-VIS spectra of standards. Upon successful spectral matching, confirmation was achieved by spiking with the corresponding standards to ensure complete identification through a peak purity test. Peaks that did not meet these criteria were excluded from quantification. Quantification was carried out using external calibration with the following standard concentrations:

- Chlorogenic acid: 0.45 mg/mL;
- Protocatechuic acid: 0.52 mg/mL;
- Delphinidin-3-*O*-glucoside: 0.39 mg/mL;
- Hyperoside: 0.40 mg/mL;
- Cyanidin-3-*O*-galactoside: 0.42 mg/mL;
- Cyanidin-3-*O*-glucoside: 0.42 mg/mL;
- Procyanidin B2: 0.36 mg/mL;
- Quercetin-3-*O*-glucoside: 0.39 mg/mL;
- Rutin: 0.48 mg/mL;
- Quercitrin: 0.52 mg/mL;
- Epicatechin: 0.40 mg/mL.

The final results were expressed as mg/g of dried drug weight.

### 3.5. Determination of Anti-Tyrosinase Activity

The anti-tyrosinase activity of the extract was assessed following a modified spectrophotometric method, which measures the diphenolase activity of mushroom-derived tyrosinase using L-DOPA as a substrate. In a 96-well microtiter plate, 50 µL of 2 mM L-DOPA prepared in phosphate buffer (50 mM, pH 6.8), 50 µL of phosphate buffer (50 mM, pH 6.8), and 50 µL of the extract solution (also in phosphate buffer, 50 mM, pH 6.8, concentration range 0.5–20 mg/mL) were added to each well. After incubation at 30 °C for 5 min, 50 µL of tyrosinase solution (250 U/mL) was added to each well. The reaction mixture was then incubated for 10 min at 37 °C. Enzymatic activity was determined by measuring absorbance at 492 nm (A_S_). The blank consisted of the same components with the extract replaced by corresponding solvent and tyrosinase replaced by buffer. The control (A_C_) contained buffer in place of the extract only. L-ascorbic acid was used as positive control, while pure solvents (W, E, TS, CS, MG) were used as negative controls for the corresponding extract to determine whether the observed bioactivity was due to the solvent itself or the extract. This was important since NaDES solvents (TS, CS, MG) are composed of substances known to possess cosmetic activity.

The tyrosinase inhibitory activity was calculated using Equation (2):

(2) $$\%\;tyrosinase\;inhibition=\frac{Ac-As}{Ac}\times 100$$

The results were expressed as IC_50_ values (mg/mL), i.e., the concentration of extract that led to 50% of tyrosinase inhibition.

### 3.6. Determination of Anti-Hyaluronidase Activity

The anti-hyaluronidase activity of the extract was evaluated using a turbidimetric 45 min method proposed by the Sigma-Aldrich protocol and used by authors [65,66] with minor modifications. Dilutions of the extracts were prepared in buffer (concentration range 0.5–20 mg/mL), and the pH of all extract solutions was adjusted to 6.4 using NaOH. The reaction mixture consisted of 50 µL of the extract solution and 100 µL of hyaluronidase enzyme solution (12.44 U/mL, dissolved in 20 mM phosphate buffer, pH 7.0). Following 10 min incubation at 37 °C, 100 µL of hyaluronic acid solution (0.3 mg/mL, dissolved in 300 mM phosphate buffer, pH 5.3) was added to the mixture. The samples were then incubated for an additional 45 min at 37 °C to allow enzymatic degradation of hyaluronic acid. After the incubation period, 1 mL of acidic albumin solution in acetate buffer (0.1% albumin, pH 3.75) was added to precipitate the remaining hyaluronic acid. Therefore, the concentration of undegraded hyaluronic acid is proportional to hyaluronidase inhibition. The absorbance of the samples (A_S_) was measured at 600 nm using an ELISA reader. The blank consisted of the same components with the extract replaced by the corresponding solvent and enzyme replaced by buffer. As controls (A_C_), a reaction mixture containing buffer in place of both the extract and enzyme were used. Tannic acid served as a positive control, while pure solvents (W, E, TS, CS, MG) were used as negative controls for the corresponding extract to determine whether the observed bioactivity was due to the solvent itself or the extract. The percentage of inhibition was calculated using the following, Equation (3):

(3) $$\%\;hyaluronidase\;inhibition=\frac{As}{Ac}\times 100$$

The results were expressed as IC_50_ values (mg/mL), i.e., the concentration of extracts that led to 50% of hyaluronidase inhibition.

### 3.7. Determination of Anti-Collagenase Activity

The anti-collagenase activity of the extract was determined according to the method described by Zhang et al. [45], which is based on the spectrophotometric quantification of hydrolytic products released from collagenase substrates (collagen or gelatin) using a ninhydrin reagent. The ninhydrin reagent was freshly prepared prior to use by mixing equal volumes of tin(II) chloride solution (1.6 mg/mL in 0.2 M citrate buffer, pH 5) and a ninhydrin solution (50 mg/mL in DMSO). The ninhydrin reagent reacts with free amino acids released from gelatin hydrolysis, forming a detectable complex whose absorbance can be measured at 545 nm. Inhibition of collagenase activity reduces the degradation of gelatin, resulting in a lower release of free amino acids, and therefore a reduced formation of the ninhydrin–amino acid complex, which is reflected by lower absorbance values.

The procedure for determining the anti-collagenase activity of the extract was based on mixing 40 µL of extract solution (at various dilutions concentration range 0.5–20 mg/mL) with 20 µL of collagenase solution (1 mg/mL, dissolved in reaction buffer: 50 mM Tris-HCl, 5 mM CaCl_2_, 1 µM ZnCl_2_, pH 7.5) and incubating the mixture at room temperature for 5 min. Then, 30 µL of gelatin substrate solution (2 mg/mL in the same reaction buffer) was added to initiate hydrolysis. Following 10 min incubation at 37 °C, 90 µL of the termination buffer (containing 120 mg/mL PEG 4000 and 25 mM EDTA) was added. Subsequently, 90 µL of ninhydrin reagent was added, and the reaction mixture was heated at 80 °C in a water bath for 10 min. After cooling to room temperature, 100 µL of distilled water was added to the reaction mixture, and absorbance was measured at 545 nm (A_S_). The blank consisted of the same components with the extract replaced by corresponding solvent and enzyme replaced by buffer. The negative and positive controls (A_C_) were prepared in the same manner, except that distilled water was used instead of the extract. Rutin was used as a positive control, while pure solvents (W, E, TS, CS, MG) were used as negative controls for the corresponding extract to determine whether the observed bioactivity was due to the solvent itself or the extract.

The percentage of collagenase inhibition was calculated using the following Equation (4):

(4) $$\%\;collagenase\;inhibition=\frac{Ac-As}{Ac}\times 100$$

The results were expressed as IC_50_ values (mg/mL), i.e., the concentration of extracts that led to 50% of collagenase inhibition.

### 3.8. In Vivo Safety Assessment

The extracts identified as those with best characteristics were evaluated on the skin of healthy volunteers. For the in vivo testing on human subjects, approval was obtained from the Ethics Committee of the Faculty of Medicine, University of Niš (approval number 12-10650/2-6 dated 3 October 2022). The participants were fully informed about the course of the study and signed written informed consent. The study was conducted in accordance with the Declaration of Helsinki. All participants were healthy volunteers with no prior history of dermatological conditions. They were instructed not to use any other cosmetic products on the tested skin area for one week prior to and throughout the in vivo testing period.

The in vivo testing was conducted using the Multi Probe Adapter System MPA^®^ 9, manufactured by Courage + Khazaka electronic GmbH, Cologne, Germany, consisting of the different probes (Corneometer^®^ CM 825, Tewameter^®^ TM 300, Mexameter^®^ MX 18 and Skin-pH-Meter^®^ PH 905). All probes were calibrated according to the manufacturer’s instructions.

The safety profile assessment of the selected extracts was conducted in a double-blind study which involved 20 healthy volunteers of both gender (25.85 ± 4.74 years). After applying the square filter paper soaked with extracts (whose pH was set using NaOH at 4.5) in the amount of 0.016 g/cm^2^ to predefined sites on the volar side of the forearm of each subject, those sites were covered with an occlusive film (Parafilm^®^, American National Can. Co., Chicago, IL, USA) and subsequently secured with self-adhesive tape (Omnifix^®^ E, Hartmann, Heidenheim, Germany). One site was left as a non-treated control without occlusion (NC), and one site was marked as non-treated control under occlusion (NCO, a site where no extract was applied, but which was still covered with the occlusive film and adhesive tape).

The following parameters were measured: EC (stratum corneum hydration), TEWL (transepidermal water loss), EI (erythema index), and pH, both before the study began (baseline values) and 60 min after the removal of occlusion, which lasted 24 h. The results were presented as mean values ± standard deviation.

### 3.9. Statistical Analysis

The IC_50_ values were presented as average result of three measurements ± standard deviation. The statistical analysis was conducted using software SPSS Statistics version 20 (IBM). Homogeneity of variances was checked with Levene’s test. For analyzing the influence of extraction solvent or the herbal material on the results, the two-way analysis of variance ANOVA was used with post hoc analysis while Welch–ANOVA was used for unequal variances. Student *t*-test was used for comparing measured in vivo parameters before and after 24 h of occlusion. The statistically significant differences were considered at *p* < 0.05. The Pearson and Spearman correlation analysis were used for comparing correlation between results, depending on whether data meet the assumptions of normality and linearity. The Shapiro–Wilk test was used for testing normality while linearity was tested using scatterplots. All the graphs and tables in the paper were constructed using Microsoft Office Excel 10 software.

## 4. Conclusions

The results of our study demonstrated better inhibitory activity of NaDES extracts against some important skin enzymes (tyrosinase, hyaluronidase, and collagenase) compared to water and ethanol extracts. In addition, these extracts also exhibited higher UV protective activity (higher SPF). The most potent extract was bilberry leaves extract made with malic acid–glycerol, which exhibited strong anti-tyrosinase (IC_50_ = 3.52 ± 0.26 mg/mL), anti-hyaluronidase (IC_50_ = 3.23 ± 0.30 mg/mL), and anti-collagenase (IC_50_ = 1.84 ± 0.50 mg/mL) activities.

In addition, this study highlighted that bilberry leaves are mostly treated as waste products in bilberry processing, exhibiting significant potential as a valuable source of active dermocosmetic ingredients, being more active than bilberry fruit extracts.

Furthermore, correlation analysis revealed correlation between UV protective and anti-tyrosinase activity, UV protective and anti-collagenase activity as well as between anti-hyaluronidase and anti-collagenase activity. UV protective and anti-tyrosinase activity also significantly correlated with the content of both chlorogenic acid and hyperoside in extracts.

The non-invasive in vivo study conducted on human volunteers demonstrated that the selected NaDES extracts, when applied under occlusion for 24 h, did not alter the basal values of skin parameters (pH, TEWL, EC, and EI).

Bearing in mind the results of our preliminary in vitro study, bilberry leaves extract prepared with malic acid–glycerol mixture have potential for use in cosmetic and/or dermopharmaceutical anti-aging products as a natural and green raw material obtained from the waste of bilberry processing in the food industry. However, further in vivo studies need to be conducted with the aim of assessing the effects of selected extracts on the skin.

## Figures and Tables

**Table 1:** Phenolic compounds identified in the analyzed extracts using HPLC. (Within each row, means labeled with different letters (e.g., a, b, c, d, e) differ significantly at *p* < 0.05. Means that share the same letter are not significantly different from one another).

Phenolic Compounds (mg/g)	TS-BF	CS-BF	MG-BF	E-BF	W-BF
Chlorogenic acid	1.53 ± 0.11 ^a^	1.51 ± 0.01 ^a^	1.47 ± 0.37 ^a^	0.83 ± 0.03 ^b^	0.86 ± 0.06 ^b^
Protocatechuic acid	1.48 ± 0.39 ^ab^	1.30 ± 0.28 ^ab^	1.35 ± 0.15 ^ab^	1.03 ± 0.02 ^ab^	0.94 ± 0.04 ^b^
Delphinidin-3-*O*-glucoside	1.07 ± 0.02 ^a^	0.99 ± 0.05 ^a^	1.01 ± 0.05 ^a^	tr	tr
Hyperoside	0.49 ± 0.01 ^a^	0.50 ± 0.07 ^a^	0.57 ± 0.06 ^a^	0.53 ± 0.03 ^a^	0.15 ± 0.03 ^b^
Cyanidin-3-*O*-glucoside	0.19 ± 0.01 ^a^	0.18 ± 0.01 ^a^	0.19 ± 0.02 ^a^	tr	tr
Cyanidin-3-*O*-galactoside	0.10 ± 0.04 ^a^	0.11 ± 0.03 ^a^	0.10 ± 0.03 ^a^	tr	tr
**Phenolic Compounds (mg/g)**	**TS-BL**	**CS-BL**	**MG-BL**	**E-BL**	**W-BL**
Chlorogenic acid	19.48 ± 0.92 ^ab^	22.48 ± 1.57 ^ab^	21.17 ± 1.75 ^ab^	22.51 ± 0.50 ^a^	8.37 ± 0.06 ^c^
Procyanidin B2	7.57 ± 1.16 ^a^	15.77 ± 0.68 ^b^	18.59 ± 1.23 ^c^	14.41 ± 0.80 ^b^	nd
Quercetin-3-*O*-glucoside	6.76 ± 0.16 ^a^	8.29 ± 0.12 ^b^	8.02 ± 0.08 ^b^	9.58 ± 0.22 ^c^	4.88 ± 0.20 ^d^
Rutin	3.16 ± 0.05 ^a^	3.60 ± 0.26 ^bc^	3.24 ± 0.10 ^ab^	3.79 ± 0.12 ^c^	1.93 ± 0.15
Hyperoside	2.21 ± 0.07 ^a^	2.85 ± 0.09 ^b^	2.57 ± 0.11 ^c^	3.31 ± 0.10 ^d^	1.32 ± 0.11 ^e^
Quercitrin	0.69 ± 0.04 ^ab^	0.90 ± 0.06 ^cd^	0.76 ± 0.05 ^ad^	0.99 ± 0.10 ^c^	0.56 ± 0.07 ^be^
Epicatechin	0.51 ± 0.09 ^a^	1.74 ± 0.08 ^b^	1.61 ± 0.27 ^b^	1.92 ± 0.13 ^b^	nd

**Table 2:** Anti-collagenase activity (IC_50_ values) of tested bilberry fruits (BF) and leaves (BL) extracts prepared using NaDES as extraction solvents (TS, CS, MG) and water (W) and 50% ethanol (E) as conventional solvents. (Different letters indicate statistically significant differences (*p* < 0.05). Extracts that share the same letter are not significantly different from each other. n.d. is an abbreviation for not detected).

IC_50_ (mg/mL)	BF	BL	Solvents (Negative Control)
TS	3.20 ± 0.03 ^ab^	2.05 ± 0.40 ^d^	n.d.
CS	3.67 ± 0.25 ^bc^	2.63 ± 0.14 ^ad^	n.d.
MG	3.02 ± 0.36 ^c^	1.84 ± 0.50 ^e^	n.d.
W	>10 ^f^	>10 ^f^	n.d.
E	>10 ^f^	>10 ^f^	n.d.
Rutin	0.35 ± 0.13 ^g^ (positive control)		

**Table 3:** Correlation matrix (** means *p* < 0.01, * means *p* < 0.05).

SPF	1.000					
Tyr	−0.879 **	1.000				
Hyal	−0.321	0.322	1.000			
Col	−0.733 *	0.512	0.817 **	1.000		
Chlorogenic acid	0.894 **	−0.759 *	0.066	−0.201	1.000	
Hyperoside	0.935 **	−0.752 *	−0.044	−0.289	0.983 **	1
	SPF	Tyr	Hyal	Col	Chlorogenic acid	Hyperoside

The color gradient reflects the strength and direction of the correlation ranging from red (strong positive correlation, r = 1) through white (no correlation, r = 0) to blue (strong negative correlation, r = −1).

**Table 4:** Chromatographic conditions for HPLC analysis of anthocyanins, flavonoids, and phenolcarboxylic acids.

Chromatographic Conditions	Anthocyanins Analysis	Flavonoids and Phenolcarboxylic Acids Analysis
Column	Lichrospher 100 RP-18e column (250 × 4.6 mm, 5.0 μm particle size)	
Mobile phase	0.1 M phosphoric acid (phase A) pure acetonitrile (phase B).	
Gradient program	0–11% B (5 min), 11–15% B (25 min), 15–18% B (8 min), isocratic 18% B (8 min), 18–30% B (4 min), 30–100% B (3 min), 100% B (7 min)	11–25% B (35 min), 25–40% B (20 min), 40–65% B (5 min), 65–100% B (10 min)
Total run time	60 min	70 min
Flow rate	0.8 mL/min	1.0 mL/min
Injection volume	4 μL	10 μL
Column temperature	25 °C	25 °C
λ for PDA detector	520 nm	260 nm

## Data Availability

The original contributions presented in this study are included in the article. Further inquiries can be directed to the corresponding author.
